# Effectiveness of Ultrasound-Guided Peritendinous Injection Treatment with Low Molecular Weight Hyaluronic Acid in Patients with Supraspinatus Tendinopathy

**DOI:** 10.3390/jcm14176291

**Published:** 2025-09-05

**Authors:** Francesco Agostini, Alessandro de Sire, Alessio Savina, Giovanni Iudicelli, Andrea Fisicaro, Giacomo Camponogara, Marco Narciso, Alessio Fricano, Marco Conti, Umile Giuseppe Longo, Valter Santilli, Antonio Ammendolia, Massimiliano Mangone, Marco Paoloni

**Affiliations:** 1 Department of Anatomy, Histology, Forensic Medicine and Orthopedics, Sapienza University, 00185 Rome, Italy; francesco.agostini@uniroma1.it (F.A.); alessio.savina@uniroma1.it (A.S.); giovanni.iudicelli@uniroma1.it (G.I.); andrea.fisicaro@uniroma1.it (A.F.); giacomo.camponogara@uniroma1.it (G.C.); marco.narciso@uniroma1.it (M.N.); alessio.fricano@uniroma1.it (A.F.); marco.conti@uniroma1.it (M.C.); massimiliano.mangone@uniroma1.it (M.M.); marco.paoloni@uniroma1.it (M.P.); 2 Physical and Rehabilitative Medicine Division, Department of Medical and Surgical Sciences, University of Catanzaro “Magna Graecia”, 88100 Catanzaro, Italy; ammendolia@unicz.it; 3 Research Center on Musculoskeletal Health, MusculoSkeletalHealth@UMG, University of Catanzaro “Magna Graecia”, 88100 Catanzaro, Italy; 4 Fondazione Policlinico Universitario Campus Bio-Medico, Via Alvaro del Portillo 200, 00128 Roma, Italy; 5 Research Unit of Orthopaedic and Trauma Surgery, Department of Medicine and Surgery, Università Campus Bio-Medico di Roma, Via Alvaro del Portillo 21, 00128 Roma, Italy

**Keywords:** tendinopathy, ultrasound-guided peritendinous injection, hyaluronic acids, supraspinatus tendon, rehabilitation

## Abstract

**Background/Objectives**: Tendinopathies represent a prevalent musculoskeletal condition characterized by load-dependent pain, stiffness, weakness, and impaired functionality. Current treatment includes therapeutic exercise, physical modalities and injective therapy. Hyaluronic acid (HA) is a fundamental component of the extracellular matrix and plays a crucial role in tissue hydration, elasticity, and lubrication. This study aims to evaluate the effectiveness of ultrasound-guided injections of HA in improving pain symptoms and functionality in patients with supraspinatus tendinopathy. **Methods**: Patients with a confirmed diagnosis of supraspinatus tendinopathy, verified through ultrasound imaging, were included in the study. Patients underwent 3 ultrasound guidance injections (1/week) of Sodium Hyaluronate (Hyalotend, 20 mg/2 mL). Patients were evaluated at the baseline, 1 month (T1), 3 months (T2), 6 months (T3), and 1 year (T4) after the first injection through the VAS, the QuickDASH and the SF-12. **Results**: Twenty-four patients were enrolled. As regards the VAS there was a statistically significant reduction (*p* < 0.01) of averages of values over time. The scores collected through the QuickDASH questionnaire have a statistically significant variation over time (*p* < 0.001). The values collected through the SF-12 Mental Component Summary (MCS) questionnaire show a highly statistically significant variation over time (*p* < 0.005). The values collected through the SF-12 Physical Component Summary (PCS) questionnaire show a statistically highly significant change over time (*p* < 0.001). **Conclusions**: Our results suggest that HA (Hyalotend) injections could represent a viable therapeutic option for patients with supraspinatus tendinopathy in the short, medium, and long term. Further studies with larger patient samples and a control group are needed to better investigate the effects and the modalities of administration of HA in tendinopathies.

## 1. Introduction

Tendinopathies represent a prevalent musculoskeletal condition characterized by load-dependent pain, stiffness, weakness of movement in the affected area, and impaired functionality [1,2]. The incidence of tendinopathies is increasing, partly due to greater participation in sports, a high number of activities exposing these structures to overload, and the aging population. The most common overuse tendinopathies involve the rotator cuff tendon, medial and lateral elbow epicondyles, patellar tendon, gluteal tendons and the Achilles tendon [3]. Tendon injuries constitute a significant percentage of musculoskeletal injuries [4]. The etiology of tendinopathy is multifactorial. A primary factor is the imbalance between the load demands placed on the tendon and its capacity to remodel. This imbalance can result from repetitive microtrauma, functional overload, or acute traumatic events [5]. At the pathophysiological level, an imbalance between collagen synthesis and degradation, hypoxia, and inflammation is observed. The tendon responds to both overload and inactivity with acute and chronic adaptations, modifying metabolism, vascularization, and the composition of the extracellular matrix [4,5,6]. Tendon pathology typically presents with a loss of normal collagen architecture due to an imbalance between collagen synthesis and degradation by metalloproteinases (MMPs). An increase in the production of type III collagen and a decrease in the synthesis of type I collagen are observed [4,5]. Tendons subjected to overload and maximal tension can undergo ischemic processes, followed by reperfusion during the relaxation phase, leading to the production of free radicals (ROS) that can weaken the tendon structure and predispose to tendinopathy [5,6]. Hypoxia induced by ischemia causes tenocyte apoptosis, VEGF (Vascular Endothelial Growth Factor) production with consequent neoangiogenesis, and MMP production, leading to weakening of the architecture [6]. Rotator cuff tendinopathies are particularly common in athletes involved in throwing sports and in the general population [7]. In most cases, these pathologies are primarily due to degeneration of the musculo-tendinous structure, associated or not with the aging process [4,8]. Current treatment options for tendinopathies include therapeutic exercise, physical modalities (Focused Extracorporeal Shockwave Therapy, Therapeutic Ultrasound and Laser) and injective therapy (corticosteroids, Hyaluronic acid and Platelet-Rich Plasma) [8,9].

Hyaluronic acid (HA) is a natural polysaccharide, a glycosaminoglycan, abundant in connective tissues and synovial fluid [10,11]. It is a fundamental component of the extracellular matrix and plays a crucial role in tissue hydration, elasticity, and lubrication and various chronic pathologies can alter its concentration [12,13]. HA has gained increasing attention in the treatment of tendinopathies due to its lubricating properties thanks to intrinsic ability of retain water molecules [14,15]. HA can increase the viscosity and lubrication, reducing friction and improving tendon gliding within their sheaths. This can help in restoring tendon physiological conditions [15]. In addition to the temporary restoration of peritendinous space lubrication and sliding, exogenous hyaluronic acid shields tendons helping to restore physiological mechanical stimuli [16].

Thus, according to the present literature, by the present study we aimed to evaluate the effectiveness of ultrasound-guided infiltrative treatment with low molecular weight (500–730 Kda) hyaluronic acid (Hyalotend) in improving pain and functional outcomes in patients with supraspinatus tendinopathy.

## 2. Materials and Methods

This study assessed the effectiveness of low molecular weight (500–730 Kda) HA (Hyalotend) in patients with supraspinatus tendinopathies. The study protocol was submitted, reviewed, and approved by the Ethical Committee of the “Sapienza” University of Rome, Italy (protocol number 251/2020, approval no. 5940, date 23 April 2020). Outpatients in the Physical and Rehabilitation Department of the Umberto I University Hospital of Rome between September 2021 and May 2022 were screened and evaluated for inclusion. Patients with a confirmed diagnosis of supraspinatus tendinopathy, verified through ultrasound imaging and clinical examination, were enrolled in the study, whereas those with alternative potential sources of shoulder pain (e.g., tendon tears, bursitis, intra-articular pathology) were excluded. The study was conducted in accordance with the Declaration of Helsinki and in compliance with Good Clinical Practice guidelines. Results have been reported following the Consolidated Standards of Reporting Trials (CONSORT) guidelines [17].

### 2.1. Study Population

To be included in the study, patients had to meet the following inclusion criteria:

- pain for at least 4 weeks
- diagnosis of tendinopathy, confirmed by magnetic resonance imaging (MRI) and/or standard ultrasound examination
- Visual Analogue Scale (VAS) of at least 2 points

Exclusion Criteria were the following ones:

- corticosteroid or local anti-inflammatory injections or were on oral corticosteroid therapy within four weeks before the treatment
- shockwave therapy six months before the treatment
- surgery in the previous twelve months
- acute post-traumatic symptoms
- advanced osteoarthritis
- calcific tendinopathy
- infections or skin diseases near the affected area
- hypersensitivity to the components of the drug
- uncontrolled diabetes mellitus, vasculopathy, peripheral neuropathy, neurological, and/or rheumatological diseases

### 2.2. Study Procedures and Evaluations

Patients who met the inclusion and exclusion criteria and provided informed consent were enrolled in the study and registered in the database at baseline. All patients were recruited from the physiatrist clinic at our hospital. At enrollment, a comprehensive objective examination was performed including ultrasound examination (XCube70, Alpinion) of the shoulder was performed, in order to exclude other possible causes of pain. The ultrasound probe used for both ultrasound assessment and ultrasound-guided injections was the linear array transducer (L10-25H), which supports a frequency range of 10–25 MHz. After ultrasound examination, patients underwent 3 injections (1/week) and all enrolled patients didn’t undergo any other treatments except for the injections.

We adhered to a treatment protocol employing the same formulation of hyaluronic acid, with identical dosage and route of administration as reported in previously published studies [6,18].

Each injection followed the same protocol, performed by a single physiatrist, an expert on this field. During the injection procedure, patients were positioned supine with the affected arm placed under the gluteus to expose the supraspinatus tendon. Any potential adverse effects were monitored during the procedure. The procedure employed a prefilled syringe of Hyalotend. The product used in the study was Sodium Hyaluronate (Hyalotend, 20 mg/2 mL), an injectable solution specifically designed for peritendinous use. Hyalotend is a viscous solution of sodium hyaluronate (500–730 kDa) obtained through bacterial fermentation, and is provided in a sterile, prefilled syringe. The syringe was attached to a 23G needle (0.60 × 30 mm), and the injection was then performed under ultrasound guidance. The injection was performed at the pre-insertional area of supraspinatus tendon.

The injection was performed following the procedure described by Cipolletta et al. [19]. A medicated bandage was applied, and the patient was instructed to perform passive movements of the affected limb. The patient was then discharged. During the injection procedure, patients were positioned supine, with the affected arm placed beneath the ipsilateral gluteal region to optimize exposure of the supraspinatus tendon. Any potential adverse effects were closely monitored throughout the procedure [19]. The intervention employed a prefilled syringe of Hyalotend. The product used in the study was Sodium Hyaluronate (Hyalotend, 20 mg/2 mL), an injectable solution specifically designed for peritendinous administration. Hyalotend is a sterile, viscous formulation of sodium hyaluronate (500–730 kDa), produced through bacterial fermentation and supplied in a prefilled syringe. The syringe was fitted with a 23G needle (0.60 × 30 mm), and the injection was performed under continuous ultrasound guidance, targeting the pre-insertional area of the supraspinatus tendon. Prior to injection, a detailed ultrasonographic evaluation of the affected tendon was conducted. Static scanning was initially performed to assess tendon morphology and detect structural alterations consistent with degenerative tendinopathy. This was followed by dynamic scanning in real time, allowing functional assessment of tendon kinematics during movement. Following imaging assessment, the supraspinatus tendon thickness and the superior limiting interface were accurately identified under ultrasound visualization. The needle was advanced with precision until its tip reached the superficial plane adjacent to the superior limit of the degenerated tendon fibers [19]. At this point, the hyaluronate solution was slowly injected under real-time sonographic monitoring, enabling direct visualization of peritendinous distribution and progressive mechanical loosening of the tendon from the superior limiting interface. Upon completion of the infiltration, the needle was carefully withdrawn, maintaining aseptic technique throughout the procedure [19,20].

#### Follow-Ups

Patients were re-evaluated at 1 month (T1), 3 months (T2), 6 months (T3), and 1 year (T4) after the first injection. At each follow-up visit and prior to every injection, a comprehensive joint assessment was performed, including clinical and ultrasonographic evaluation, supplemented by inspection of the overlying skin for possible hematoma formation.

### 2.3. Primary Objectives

The primary endpoints were upper limb function, assessed using the QuickDASH score, and pain intensity, measured by the Visual Analogue Scale (VAS).

To evaluate the recovery of daily activities limited by upper limb tendinopathy, QuickDASH (Disabilities of the Arm, Shoulder, and Hand) scale was assessed [21].

Pain progression was monitored using the Visual Analogue Scale (VAS) to assess the reduction of pain following HA injections (500–730 kDa), administered via ultrasound-guided peritendinous injections in patients with upper limb tendinopathies [22].

### 2.4. Secondary Objectives

Secondary outcomes included health-related quality of life, evaluated through the SF-12 questionnaire, and safety of the intervention.

SF-12 (Short Form 12) Health Survey was used to assess whether the treatment impacted on the patient’s perception of their overall health status, both physical and mental [23].

About safety, the injections were carried out under strict safety, precision, and sterility conditions, in accordance with methodologies reported in authoritative scientific literature [19,24]. At each follow-up, the possible appearance of adverse effects related to the procedure or to the use of HA was investigated [25,26].

All outcomes, both primary and secondary, will be evaluated at the baseline visit (T0) and at follow-up visits at 4 weeks (T1), 12 weeks (T2), 6 months (T3), and 12 months (T4) after the end of the treatment.

### 2.5. Statistical Analysis

The VAS scores, QuickDASH scores, SF-12 scores were analyzed over time to determine if there were any statistically significant differences between the baseline values (T0) and follow-up values (T1, T2, T3, T4) through Friedman repeated measures analysis with subsequent post-hoc analysis. As the Friedman test was statistically significant (*p* < 0.05), we can investigate further with post hoc tests to determine where exactly the differences between time lie. Therefore, we show these pairwise comparisons. A 95% confidence interval was calculated using an appropriate binomial method. The significance value was set at 0.05. Data collection, organization, and charting were done using Excel, while the *T*-test was performed using SPSS. Version 31 For Minimally Clinically Important Differences (MCID), we drew inspiration from relevant publications on the subject [27,28,29].

#### Sample Size Calculation

The sample size was calculated using the GPower version 3.1.9.7 program. Considering the Quick Dash as the primary outcome of the study and inserting, in agreement with Franchignoni et al. [29], a *p* value of 0.05 and a power value of 0.95 as the pre-treatment value (49 ± 18) and post-treatment value (31 ± 18), an effect size of d = 1 was obtained; the sample size value was 13. The calculated effect size is in line with that reported by Chester et al. [28].

## 3. Results

The total number of patients enrolled was 28, subsequently 4 left the study for personal reasons, thus reaching a total number of patients treated and subjected to follow-up of 24. Demographic and clinical characteristics of patients included were summarized in Table 1.

### 3.1. QuickDASH Scale

**Figure 1:**
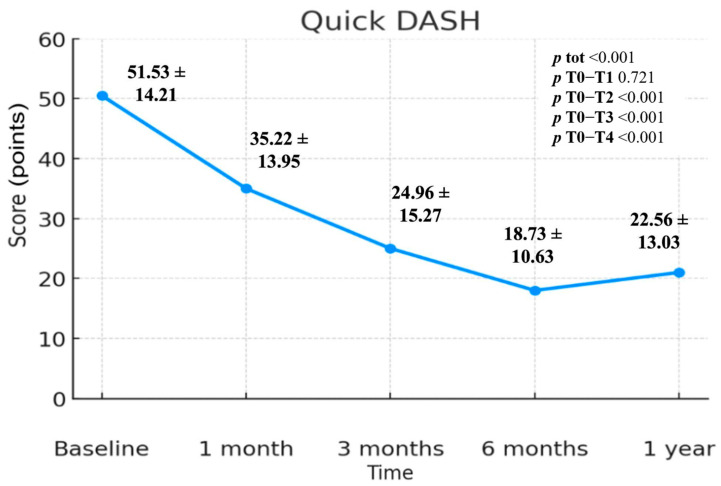
QuickDASH scale includes standard deviation values and *p*-values.

The values collected through the QuickDASH questionnaire have an extremely statistically significant variation over time (*p* < 0.001). The change is statistically significant compared to T0–T2 (*p* < 0.001), T0–T3 (*p* < 0.001) and T0–T4 (*p* < 0.001). There is therefore no statistically significant variation in the T0–T1 interval (*p* = 0.721). The values were 51.53 (T0), 35.22 (T1), 24.96 (T2), 18.73 (T3) and 22.56 (T4); (Figure 1).

Comparing the values measured between T0 and T4 we find a reduction of 28.97 value. For this scale, the greatest reduction occurred between T0 and T3 with 32.8 means points. In Figure 1 there is a constant reduction in QuickDASH value over time up to T3 then a rise in the period between T3 and T4. The difference between T0 and T4 sequel is one reduction of 28.97 means value. (Figure 1; Table 2)

### 3.2. Visual Analogue Scale (VAS)

**Figure 2:**
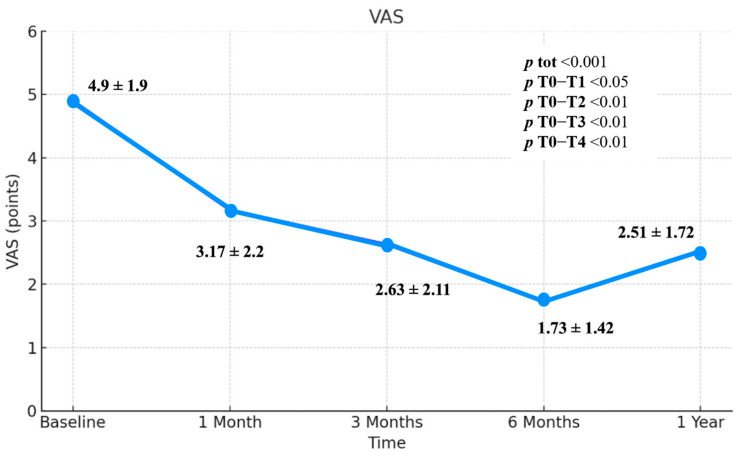
Visual Analogue Scale (VAS), 0–10 points, includes standard deviation values and *p*-values.

As regards VAS, there was a statistically significant reduction (*p* < 0.01) of values over time. There was a statistically significant reduction in VAS from T0 (mean = 4.9) to T4 (mean = 2.51) (*p* < 0.01). The reduction in VAS is also statistically significant when comparing the scores obtained at the T0 and T1 (*p* < 0.05), T0–T2 (*p* < 0.01) and T0–T3 (*p* < 0.01). The means are respectively 4.9 points (T0), 3.17 points (T1), 2.63 points (T2), 1.73 points (T3), and 2.51 points (T4) (Figure 2). The lowest value was therefore found at T3 after the injections cycle. There is an increase of 0.78 points between the value at T3 and those at T4. The difference between T0 and T4 is instead 2.39 points. In Figure 1 there was therefore a reduction in VAS values over constant time up to T3 and then a rise in the period between T3 and T4. The difference between T0 and the last follow-up is a reduction of 2.39 points. (Figure 2; Table 2).

### 3.3. SF-12 Scale

#### 3.3.1. SF-12 Physical Component Summary (PCS)

**Figure 3:**
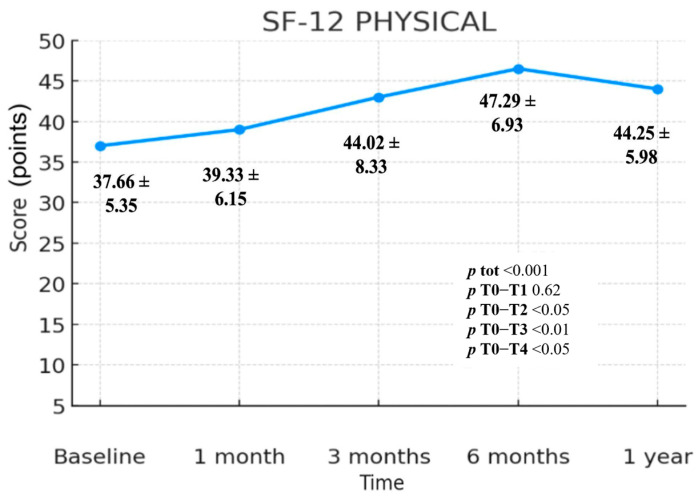
SF-12 scale—Physical Component Summary (PCS) index, includes standard deviation values and *p*-values.

The values collected through the SF-12 Physical Component Summary (PCS) questionnaire show a statistically highly significant change over time (*p* < 0.001). The change is statistically significant in the comparisons T0–T2 (*p* < 0.05), T0–T3 (*p* < 0.01) and T0–T4 (*p* < 0.05), while no statistically significant difference is observed in the interval T0–T1 (*p* = 0.62). The mean scores were 37.66 (T0), 39.33 (T1), 44.02 (T2), 47.29 (T3) and 44.25 (T4); (Figure 3). The comparison of the values recorded at T0 and T4 reveals an overall increase of 6.59 points. The greatest improvement for this scale occurs between T0 and T3, with a gain of 9.63 mean points. A steady increase in SF-12 PCS values up to T3 is evident, followed by a modest decline between T3 and T4, which however remains well above baseline. The difference between T0 and T4 therefore represents a net increase of 6.59 mean points. (Figure 3; Table 2)

#### 3.3.2. SF-12 Mental Component Summary (MCS)

**Figure 4:**
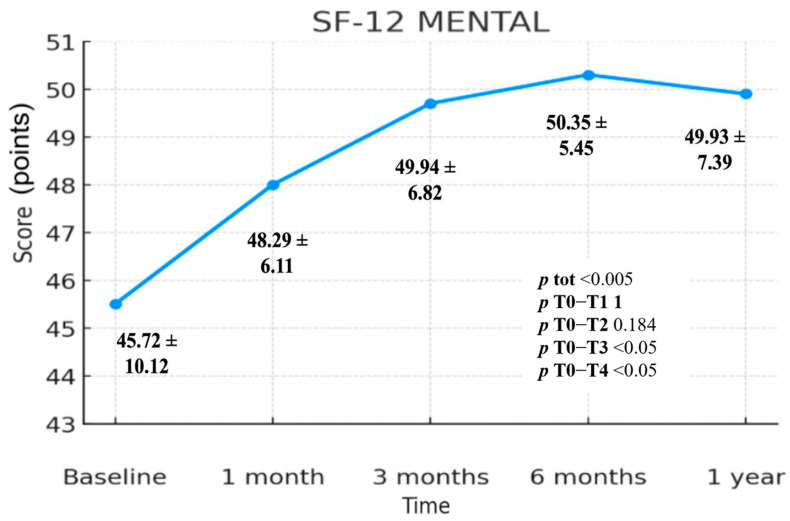
SF-12 scale—Mental Component Summary (MCS) index, includes standard deviation values and *p*-values.

The values collected through the SF-12 Mental Component Summary (MCS) questionnaire show a highly statistically significant variation over time (*p* < 0.005). The change is statistically significant in the T0–T3 (*p* < 0.05) and T0–T4 (*p* < 0.05) comparisons, whereas no statistically significant differences are observed in the T0–T1 (*p* = 1.000) and T0–T2 (*p* = 0.184) intervals. The mean scores were 45.72 (T0), 48.29 (T1), 49.94 (T2), 50.35 (T3) and 49.93 (T4), (Figure 4). Comparing T0 and T4, we observe an overall increase of 4.21 points. The greatest change for this scale occurs between T0 and T3, with a gain of 4.63 mean points. In Figure 3 a steady rise in SF-12 MCS values is evident up to T3, followed by a slight decline between T3 and T4 that nonetheless remains above baseline. The difference between T0 and T4 therefore represents an overall increase of 4.21 mean points (Figure 4; Table 2).

#### 3.3.3. Safety

All interventions were executed under strictly controlled aseptic conditions, employing continuous ultrasound guidance to ensure anatomical precision and procedural reproducibility. These methods are consistent with validated protocols described in literature on musculoskeletal interventional techniques. The procedure was performed exclusively by experienced operators trained in ultrasound-guided injections, thereby minimizing procedural variability and mitigating risk. No adverse events, including infection, neurovascular compromise, or prolonged post-procedural pain, were observed during the study period. These findings reaffirm the established safety profile of ultrasound-guided peritendinous hyaluronic acid injections and support their inclusion as a low-risk therapeutic option for shoulder tendinopathies [19,24,25,26].

## 4. Discussion

The statistical analysis of our study data suggests that ultrasound-guided infiltrations of low molecular weight (500–730 kDa) HA (Hyalotend) constitute an effective therapeutic tool in the management of supraspinatus tendinopathy.

Analysis of the Quick DASH questionnaire showed a steady decline in mean scores from baseline (T0) to six months (T3), followed by a slight increase from six months to one year (T4). This finding suggests that ultrasound-guided infiltrations of low molecular weight (500–730 kDa) HA (Hyalotend) at the supraspinatus tendon level contribute to restoring upper limb function, particularly in activities of daily living. The T0 value was 51.53, while the T4 value was 22.56, showing a statistically significant difference (*p* < 0.001). Notably, no statistically significant difference was observed between T0 and T1 (*p* = 0.721), despite a value reduction of 16.31 points (from 51.53 to 35.22). The first statistically significant difference (*p* < 0.001) was identified at T2, indicating that while pain reduction occurs within the first month (T1), a clear improvement in daily function is perceived by patients after three months (T2). Similar to VAS, the greatest improvement in Quick DASH was observed at T3, with a mean difference of 32.8 points, from 51.53 to 18.73.

Observing the trend of the VAS, a statistically significant reduction (*p* < 0.001) is evident between baseline (T0) and follow-up at one year (T4). Furthermore, a statistically significant reduction (*p* < 0.05) is also observed between baseline and T1, with the VAS mean decreasing from 4.9 cm to 3.7 cm. This early pain reduction is particularly relevant for the clinician, as rapid symptom relief is crucial in pain management. Additionally, early pain reduction allows the patient to promptly engage in a broader physiotherapy program, enabling a greater range of motion and better tolerance of physical therapy sessions. The maximum reduction in VAS was observed at six months (T3), with a statistically significant decrease (*p* < 0.001), from a mean VAS of 4.9 cm to 1.73. This appears to be the time point at which patients achieved the greatest pain relief and the most substantial benefit from treatment. However, there was an increase in mean VAS scores from 1.73 cm to 2.51 between six months (T3) and one year (T4), indicating a decline in therapeutic effect over time. Nonetheless, comparing the one-year follow-up (T4) to baseline (T0), the reduction in VAS remained statistically significant (*p* < 0.001), with a mean decrease from 4.9 to 2.51, suggesting an overall sustained therapeutic effect.

The SF-12 questionnaire was analyzed in its Physical and Mental Health components. Both components followed a pattern similar to the VAS and Quick DASH, with a progressive improvement in scores until six months (T3), followed by a decline between T3 and T4. However, when comparing T4 to T0, both components showed a statistically significant difference (*p* < 0.05).

In the Mental Health component, value improved from 45.72 (T0) to 49.93 (T4), with a difference of 4.21 points (*p* < 0.05). In the Physical Health component, value increased from 37.66 to 44.25, with a difference of 6.59 points (*p* < 0.001). This suggests that patients experienced improvements in both physical and mental well-being, with a more pronounced improvement in mental health. Given that shoulder pain significantly limits daily activities and often leads to sacrifices in sports and hobbies, the recovery of such activities may explain the notable increase in the mental health score.

From a clinical point of view, the early reduction in pain observed after just one month (T1- Short-term efficacy) is very important, as it facilitates the early involvement of the patient in structured physiotherapy programs [8,20,25,30,31]. Such early intervention can accelerate functional recovery and reduce the risk of chronicity and recurrence. Therefore, HA acts as an enabling agent within a multimodal rehabilitation strategy, improving both adherence to and the effectiveness of therapeutic exercise protocols [32].

From the results, we observe a peak at six months (medium-term efficacy) and a slight decline at 12 months (long-term efficacy). This finding could help us better understand the efficacy and possible timing of retreatment. The sustained benefits observed at 12 months (long-term efficacy), together with the effective use of retreatment cycles, particularly in athletes exposed to high functional loads, further emphasize the role of (500–730 kDa) HA in the long-term management of chronic tendinopathies [25,33]. In particular, the therapeutic profile of HA is influenced more by dosage and specific formulation characteristics than by molecular weight alone, underling the importance of well-defined and standardized treatment protocols, such as the one adopted in our study [34,35].

The use of HA in tendinopathies is confirmed thanks to its properties and effects on the tendon system. First of all, it promotes lubrication and viscoelasticity, in fact, it increases the viscosity, reducing friction between the tendon and the surrounding structures and facilitating the sliding of the tendon. Secondly, the restoration of tendon physiological conditions helps in reducing pain symptomatology. Finally, it plays a role in viscosupplementation as it acts as a natural lubricant, improving tendon sliding, reducing friction and pain during movement [35].

The therapeutic efficacy of hyaluronic acid (HA) goes beyond its established role in restoring peritendinous lubrication and viscoelasticity, thereby reducing friction and facilitating tendon gliding [8]. Literature highlights its more active involvement at the cellular and molecular level. Specifically, low molecular weight HA (500–730 kDa), used in our study, has been shown in vitro to improve tenocyte viability and upregulate type I collagen expression, an effect that is particularly relevant in addressing the pathological collagen remodeling typical of tendinopathies, characterized by an increase in type III collagen production and a decrease in type I collagen production [8,13,36]. Specifically, low molecular weight HA (Hyalotend) seems to be more suitable and effective in tendinopathies reducing symptoms, improving biomechanical function [8,12,13,34,35,36].

Furthermore, HA appears to mitigate oxidative stress and apoptosis in human tenocytes by modulating critical intracellular pathways, including Nrf2 and caspases 3 and 7. These findings support the concept that HA is not only a mechanical lubricant but also an active modulator of the extracellular matrix capable of counteracting structural and cellular damage associated with mechanical overload and ischemia [37,38,39,40,41].

Our study could demonstrate that HA has a positive impact not only in terms of pain reduction but also in improving functionality in patients affected by tendinopathies. However, within the scientific community, there is no unanimous consensus on this treatment, likely due to the lack of selection of patients, different localizations (gliding tendons or anchoring tendons), different severity of the tendinopathies and different protocols of treatment with different kinds of HA [40,41]. We selected this protocol based on our clinical experience and our previous studies and reviews considering how HA has emerged as a promising therapeutic option for the conservative treatment of tendinopathies [6,8,18].

In particular, we considered the study by Osti et al. [42], which demonstrated how differences in HA preparations influence its specific mode of action in a dose-dependent rather than molecular weight-dependent manner [42,43]. Although the effects of HA in patients with tendinopathies are still partially unknown, its potential effects could lead to functional and kinematic improvements [8,44]. It is evident that individual patient characteristics play a crucial role in the efficacy of HA treatment. In this regard, Frizziero et al. [45], in 2015 conducted a study on low molecular weight HA (500–730 kDa) (Hyalotend) injections in tendon damage associated with de-training, stratifying rat tendons based on training levels. The authors had previously shown that detraining affects the patellar tendon proteoglycan content and collagen fiber organization [37]. Therefore, with the aim of improving the knowledge on the adaptation of the patellar tendon and its enthesis to detraining from a histological and histomorphometric point of view, and to investigate the hypothesis that repeated peri-patellar injections of HA on the patellar tendon can reduce detraining and limit the damage associated with detraining, they enrolled twenty-four male Sprague-Dawley rats and divided them into 3 groups: Untrained, Trained and Detrained. In the detrained rats, the left tendon was not treated, while the right tendon received repeated peri-patellar injections of HA or saline. Subsequently, the structure and morphology of the patellar tendons and enthesis were evaluated. The authors concluded that HA treatment is effective in maintaining the structural properties of the patellar tendon and enthesis and that these beneficial effects could play a significant role in conservative and rehabilitative management strategies [37]. HAs characterized by lower molecular weights (500–730 kDa) can lay the groundwork for better outcomes about the management of rotator cuff tendinopathy.

HA injections, particularly low molecular weight HA (500–730 kDa), have demonstrated efficacy in reducing pain and improving function, with an excellent safety profile and minimal side effects [8,45,46]. HA offers additional benefits such as faster action, lower costs and reduced post-injection pain, making it particularly suitable for athletes [25,33]. Ultrasound guidance is recommended to ensure precise delivery, particularly in rotator cuff tendinopathy. At the molecular level, HA can help in supporting extracellular matrix remodeling and tendon health. However, variability in HA formulations, dosages, and treatment protocols limits standardization also considering the role that might have physical exercise [6,8,34,35,39,46].

The ultrasound-guided infiltrative treatment proved effective, with benefits perceived by the patient as early as the first injection [19]. The long-term therapeutic effects are evidently influenced by the clinical condition and baseline health status of the target tendon. When the tendon is in good condition, the therapeutic effects on pain and functionality persist for extended follow-ups (>one year), particularly when combined with additional rehabilitative interventions [30,31,32,33,34].

Furthermore, satisfactory results have been achieved in cases of retreatment. These cases primarily involved athletes who, after an initial substantial benefit lasting more than six months but less than one year, underwent an additional cycle of three ultrasound-guided infiltrations with the same low molecular weight HA (500–730 kDa) formulation (Hyalotend) [6].

Across all performed injections, no adverse effects were reported. These findings underscore and highlight the safety of ultrasound-guided infiltrative treatment with low molecular weight HA (500–730 kDa) (Hyalotend) for the management of supraspinatus tendinopathy.

The absence of significant adverse events, including infections or neurovascular complications, and the precision guaranteed by continuous ultrasound guidance confirm that the ultrasound-guided technique ensures safety and accuracy.

The choice to use HA over other pharmacological injection options (such as corticosteroids), which may present greater risks, systemic side effects or degenerative effects on the tendon, has proven to be better from a safety perspective [8,47]. This makes HA a particularly attractive choice, especially for athletes, where a rapid return to activity with a minimal risk profile is a priority. The repeatability of the procedure and its performance by experienced operators are key elements of this safety [48,49].

Future research should prioritize randomized controlled trials (RCTs) comparing hyaluronic acid (HA) with placebo or alternative conservative interventions, such as physiotherapy alone, in order to accurately quantify its added value and specific treatment effects.

The accuracy of ultrasound-guided injections, performed by experienced physicians, is a critical factor in maximizing effectiveness in patients with shoulder tendinopathy. Accurate targeting minimizes procedural risks and ensures optimal delivery of the therapeutic agent, further enhancing the clinical utility of HA in the management of subjects affected tendinopathies.

### Study Limitations

We are aware that the study showed some limitations. Firstly, the main one is the lack of a control group, that might affect the results considering that the effects observed could be due to a potential placebo effect by all the treated patients. Future studies with a control group undergoing physiotherapy alone or a combination with HA injections for comparative evaluation are needed.

Furthermore, another limitation is that all our outcomes were patient-reported, without imaging outcomes such as the elastosonographic assessment of the tendon that had not be performed due to the unavailability of the required equipment. However, the integration of ultrasound elastography in future investigations may provide valuable insights into tendon-specific structural and biomechanical changes over time, potentially enhancing the understanding of tissue response to treatment.

Lastly, given that the trend of VAS, Quick DASH, and SF-12 scores begins to reverse between six months (T3) and one year (T4), extending the study’s monitoring period would be valuable to determine the long-term duration of the therapeutic benefit of low-molecular weight HA injections.

## 5. Conclusions

Taken together, findings of this study showed that patients with supraspinatus tendinopathy treated with low-molecular weight HA(Hyalotend) injections obtained pain relief and an overall improvement in functioning. The improvement appears to be prolonged and sustained over a twelve-month observation period, consistent with the reduction in patient-perceived disability reported in our results. Furthermore, no severe complications were observed in the study participants, suggesting that HA (Hyalotend) injections might represent a viable therapeutic option for conservative treatment for patients with supraspinatus tendinopathy in the short, medium, and long term. Further studies with larger patient samples and a control group are needed to better investigate the effects and the modalities of administration of HA in tendinopathies, including objective imaging findings (e.g., tendon thickness, neovascularization, etc.) over time, as this treatment continues to demonstrate promising and positive results.

Future research is needed to explore and directly compare the therapeutic outcomes of combining hyaluronic acid with conventional rehabilitation programs versus conventional rehabilitation alone, to better define its potential additive value within multidisciplinary treatment strategies.

## Figures and Tables

**Table 1:** Demographic and clinical characteristics of patients included.

Characteristics	Participants (*n* = 24)
Sex, M/F (%M)	14/10 (58.33%)
Age (Mean ± Sd)	59.7 ± 11.1 Years
BMI (Mean ± Sd)	26.95 ± 3.51 kg/m^2^
Side Affected, Left/Right (%Left)	8/16 (33.33%)
Dominant Side, Left/Right (%Left)	5/19 (20.83%)

**Table 2:** Means and standard deviations in relation to time.

	T0	T1	T2	T3	T4	*p* tot	*p* T0–T1	*p* T0–T2	*p* T0–T3	*p* T0–T4
Quick-DASH	51.53 ± 14.21	35.22 ± 13.95	24.96 ± 15.27	18.73 ± 10.63	22.56 ± 13.03	<0.001	0.721	<0.001	<0.001	<0.001
VAS	4.9 ± 1.9	3.17 ± 2.2	2.63 ± 2.11	1.73 ± 1.42	2.51 ± 1.72	<0.001	<0.05	<0.01	<0.01	<0.01
SF-12 Mental	45.72 ± 10.12	48.29 ± 6.11	49.94 ± 6.82	50.35 ± 5.45	49.93 ± 7.39	<0.005	1	0.184	<0.05	<0.05
SF-12 Physical	37.66 ± 5.35	39.33 ± 6.15	44.02 ± 8.33	47.29 ± 6.93	44.25 ± 5.98	<0.001	0.62	<0.05	<0.01	<0.05

## Data Availability

All data is available in the article.
